# Digging into the Role of Inflammatory Biomarkers in Sudden Sensorineural Hearing Loss Diagnosis and Prognosis: A Systematic Review and Meta-Analysis

**DOI:** 10.3390/medicina58070963

**Published:** 2022-07-20

**Authors:** Andrea Frosolini, Leonardo Franz, Antonio Daloiso, Andrea Lovato, Cosimo de Filippis, Gino Marioni

**Affiliations:** 1 Department of Neuroscience DNS, Audiology Unit, University of Padova, 31100 Treviso, Italy; andreafrosolini@gmail.com (A.F.); cosimo.defilippis@unipd.it (C.d.F.); 2 Department of Neuroscience DNS, Otolaryngology Section, University of Padova, 35100 Padova, Italy; leonardo.franz@aopd.veneto.it (L.F.); antoniodaloiso96@gmail.com (A.D.); 3 Guided Therapeutics (GTx) International Scholarship Program, Techna Institute, University Health Network (UHN), Toronto, ON M5G2C4, Canada; 4 Otolaryngology Unit, Vicenza Hospital, 36100 Vicenza, Italy; andrea.lovato.3@hotmail.it

**Keywords:** sudden sensorineural hearing loss (SSNHL), circulating biomarkers, tumor necrosis factor alpha, C-reactive protein, prognosis

## Abstract

*Background and Objectives*: Sudden Sensorineural Hearing Loss (SSNHL) is a quite common clinical finding in otolaryngology. Most cases are classified as idiopathic and there is a dearth of information on factors able to predict the response to treatment and hearing recovery. The main aim of this systematic review and meta-analysis was to assess and critically discuss the role of circulating inflammatory biomarkers in SSNHL. *Materials and Methods*: A search was conducted of the English literature published between 1 January 2009 and 7 July 2022 on Pubmed, Scopus, Web of Science, ScienceDirect, and Cochrane following PRISMA guidelines. *Results*: A total of 256 titles were retrieved from the search. After full-text screening and application of inclusion/exclusion criteria, 13 articles were included. Twelve out of thirteen studies reported significant differences in biomarkers values in SSNHL patients, of which Tumor Necrosis Factor alpha (TNF-α) and C-reactive Protein (CRP) were the most analyzed. Our meta-analysis for CRP’s mean values in SSNHL groups vs. controls showed significantly higher CRP levels with a pooled overall difference of 1.07; confidence interval (CI) at 95%: 0.03; 2.11. For TNF-α, discordant results were found: three studies showed significantly higher levels in SSNHL patients vs. controls, whereas other three investigations showed lower levels in the SSNHL groups (overall pooled difference 1.97; 95% CI: −0.90; 4.84). A high between-study heterogeneity was found. *Conclusions*: This systematic review pointed out that, although there exists a growing literature in the field of circulatory biomarkers identification in SSNHL, there is a high heterogeneity of results and low quality of evidence. CRP resulted to be higher in SSNHL patients than in controls, while TNF-α showed more heterogeneous behavior. The data reported herein needs to be confirmed in well-designed prospective multicenter randomized studies, with the objective of improving SSNHL treatment and outcome and thereby reducing the social burden of hearing loss.

## 1. Introduction

Sudden Sensorineural Hearing Loss (SSNHL) is described as a hearing loss of not less than 30 dB at three contiguous frequencies occurring within 72 h [1]. With a worldwide incidence ranging from 8–15 per 100,000 population a year, SSNHL is a common clinical finding in otolaryngology. Most cases of SSNHL (about 90%) are classified as idiopathic [1]. Accordingly, there is a dearth of information on factors able to predict the response to treatment and hearing recovery [2].

A diagnostic biomarker is used to detect or confirm the presence of a disease or condition of interest, whereas a prognostic biomarker is used to identify the likelihood of a clinical event, disease recurrence, and progression [3]. In recent years, given the importance of also identifying promptly-available, minimally-invasive, and inexpensive biomarkers, in SSNHL, the diagnostic and prognostic role of peripheral immune system cells such as Neutrophil count, Lymphocyte count, Neutrophil Lymphocyte ratio (NLR), and Platelet Lymphocyte ratio (PLR) have been pointed out in several investigations [4,5,6]. Moreover, a recent meta-analysis showed that a high Fibrinogen level was associated with poor prognosis in SSNHL patients [7]. Circulating inflammatory biomarkers including C Reactive Protein (CRP), Erythrocyte Sedimentation Rate (ESR) and procalcitonine, as well as immunologic parameters, interleukins, and cytokines such as Tumor Necrosis Factor alpha (TNF-α) have been studied in SSNHL with discordant results [8].

Therefore, a systematic review and meta-analysis were performed in order to assess and critically discuss the current knowledge and evidence regarding the role of circulating inflammatory biomarkers in SSNHL.

## 2. Materials and Methods

### 2.1. Electronic Database Search

A search was conducted of the English literature published in the period between 1 January 2009 and 7 July 2022 on the databases Pubmed, Scopus, Web of Science, ScienceDirect and Cochrane, following the PRISMA guidelines [9]. The following keywords were used: TNF-α; CRP; Erythrocyte Sedimentation Rate (ESR); Antinuclear antibodies (ANA); Anti-neutrophil Cytoplasm Antibodies (ANCA); procalcitonin; interleukin (IL); cytokine; sudden sensorineural hearing loss. MeSH terms and keywords were combined accordingly on the aforementioned databases. The reference lists of all the included articles were accurately screened in order to identify other pertinent studies. The “Related articles” option present on the PubMed homepage was also considered.

### 2.2. Inclusion and Exclusion Criteria

A study was included only if the following general criteria were met: (i) the investigation regarded the diagnostic and/or prognostic values of blood inflammatory markers in SSNHL; (ii) SSNHL was diagnosed as a hearing loss of not less than 30 dB occurring within 72 h; (iii) a control group of patients without SSNHL was considered; (iv) detailed information about laboratory tests were reported. Exclusion criteria were: (i) study design of case report, editorial, survey, letter to the editor and review; (ii) animal model study and (iii) non-English language paper.

### 2.3. Data Extraction and Quality Assessment

The authors analyzed the data from the available literature. Included studies were analyzed to extract all available data and assure eligibility for all patients. The Newcastle-Ottawa Scale (NOS) of studies was used to assess the quality of the investigations included [10]. Any disagreements about inclusion/exclusion of investigations were solved by a discussion among team members.

### 2.4. Statistical Analysis

A meta-analysis was conducted to compare the values of inflammatory biomarkers in SSNHL and non-SSNHL control groups. Pooled weighted mean differences (WMD) were estimated using a random-effects model. The overall effect size was estimated and forest plots. Heterogeneity was investigated by performing subgroup analysis and meta-regression. The ‘‘meta’’ suite of Stata 16 (Stata Corp., College Station, TX, USA) was used.

## 3. Results

### 3.1. Retrieving Studies

**Figure 1:**
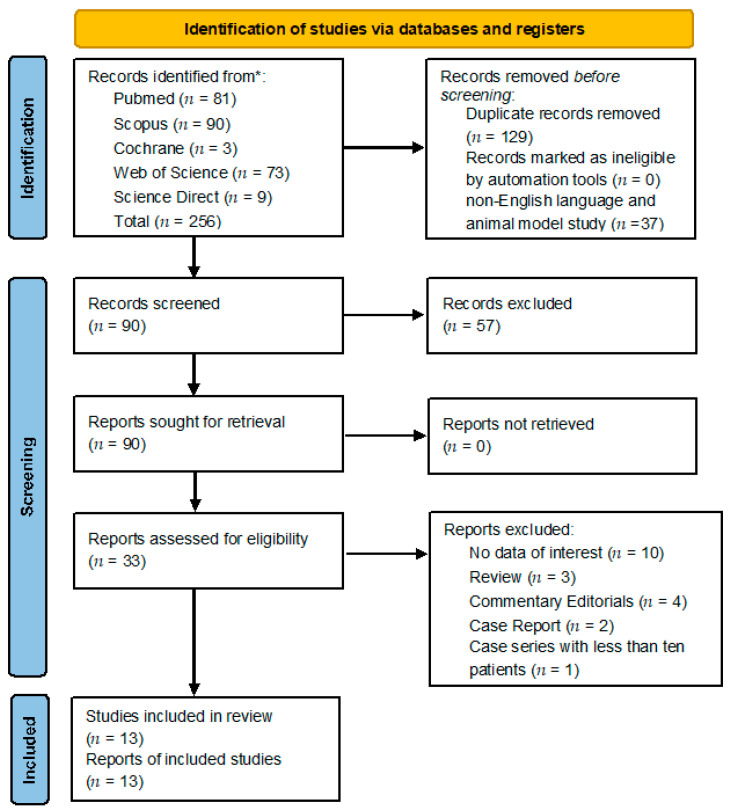
PRISMA [9] Diagram resembling Electronic Database Search and Inclusion/Exclusion process of the review. Legend: * date of last search 7 July 2022.

A total of 256 titles were retrieved from the database search and from cross references checking (81 from Pubmed; 90 from Scopus; 73 from Web of Science; 9 from ScienceDirect and 3 from Cochrane). After removal of duplicates, non-English language, and animal model study, 90 manuscripts were identified. Selection based on title and abstract screening led to the exclusion of 57 studies. The 33 remaining studies potentially relevant to the topic were accurately examined and, after full-text screening and application of inclusion/exclusion criteria, 13 articles were included in the qualitative synthesis (see Figure 1) [11,12,13,14,15,16,17,18,19,20,21,22,23]. Subsequently, 9 articles were included in the quantitative synthesis [11,13,14,15,16,19,20,22,23].

### 3.2. Assessing of Quality

All included studies had adequate relevance to the subject of this systematic review. No studies were a randomized controlled trial; five out of thirteen were retrospective studies [11,12,13,16,17] and eight prospective ones [14,15,18,19,20,21,22,23]. Randomization, concealed allocation, and baseline comparability were not achieved in company study and according to the NOS scale [10], and the quality of included case-control studies was overall moderate or high, ranging from 5 to 9 stars out of nine (see Table 1). A main issue was the poor selection of controls: only 4 studies enrolled community controls [15,17,18,22], whereas all the others selected hospital controls, with potential ongoing pathologies perhaps responsible for alterations of the studied biomarkers [11,12,13,14,16,19,20,21,23].

### 3.3. Qualitative Synthesis

The more investigated markers were CRP and CRP/albumin ratio [11,12,13,16,17,19]; TNF-α [13,14,15,19,20,22,23]; High Density Lipoprotein (HDL) and Low-Density Lipoprotein (LDL) [13,18]; Inflammatory cells Neutrophil (N), leucocyte (L) and monocytes (M) [13,16,17,19,20,21]. Other studied markers were ESR [13,21]; Anti Cyclic Citrullinated Peptides (CCP) [21]; Anti Double Strand (DS) DNA [21]; Complement (C) 3 and C4 factor [21]; Q10 Coenzyme [18]; Procalcitonin [11]; IL 6, 10 and 12 [19,23]; Cholesterol [18]; Natural Killer Cell Activity (NKCA) [19]; Cluster of Differentiation (CD) 40 and CD86 [13,20]; Nuclear Factor Kappa-light-chain-enhancer of activated B cells (NFKB) [15]; Toll Like Receptor (TLR)4 [15]; Anti-Nuclear Antibody (ANA) [14,15]; Interferon-γ [20]; Nervonic Acid [18] anti Heat Shock Protein (HSP) 70 [14]. The time intervals elapsed from the onset of SSNHL to the execution of blood sampling, reported only by 6 out of 13 research groups [12,13,16,17,19,23], are shown in Table 2 and ranged from 6 h [13] to 5 months [16].

#### 3.3.1. Biomarkers in SSNHL vs. Control Groups

Twelve out of thirteen studies reported at least one significantly higher value of analyzed biomarkers between SSNHL and non-SSNHL control groups, as summarized in Table 2 [11,12,13,14,15,16,17,18,19,20,21,22]. Demirhan et al. [23] found no significant differences in levels of TNF-α, IL-10, and IL-12 between 23 SSNHL cases and 20 healthy age-matched control subjects. Four studies found significant differences regarding TNF-α values, and in 3 studies TNF-α was higher in SSNHL compared to healthy controls with no history of hearing loss: 4.11 ± 0.91 pg/mL vs. 1.16 ± 0.31 pg/mL [15]; 15.8 ± 9.3 pg/mL vs. 12.4 ± 8.7 pg/mL [20]; 24.1 ± 3.7 pg/mL vs. 14.4 ± 1.7 pg/mL [22]; in one case [14] the TNF-α marker was higher in the control group 12.2 ± 7.9 pg/mL compared to SSNHL 11.4 ± 1.2 pg/mL.

Öçal et al. [12] found a significantly higher CRP/Albumin ratio (CAR) in the SSNHL group compared to controls (0.95 ± 0.47 vs. 0.74 ± 0.13). Three studies found significantly higher M values in SSNHL vs. control group: 308 ± 105 U/μL vs. 217 ± 58 U/μL [19]; 26.36 ± 4.3 vs. 14.32 ± 2.3 [20]; 1.41 ± 1.57 vs. 1.7 ± 1.40 [21].

Cayr et al. [17] found significantly higher values of Fibrinogen Albumin Ratio (FAR), CAR and NLR between both subgroups of SSNHL patients compounded of Good Prognosis (GP) and Poor (PP) (33 GP and 14 PP patients, respectively) vs. 41 healthy matched Control Subjects (CS). Herein are the retrieved mean values: FAR 51.56 (range 34.12–138.6) CS vs. 69.96 (43.03–134.84) GP vs. 85.22 (64.65–139.02) PP; CAR 0.5 (0.21–1.51) CS vs. 0.73 (0.19–1.64) GP vs. 0.89 (0.25–2.19) PP; NLR 1.77 (1.3–3.1) CS vs. 2 (1.6–3.5) GP vs. 2.61 (1.5–3.3) PP.

Liu et al. [15] found significantly higher NFKB (3.98 ± 0.79 μg/mL vs. 1.31 ± 0.36 μg/mL) and TLR-4 values (4.69 ± 0.92 μg/mL vs. 1.86 ± 0.49 μg/mL). Cadoni et al. [18] found a significant difference in values of Q10 (0.51 ± 0.21 SSNHL vs. 1.15 ± 0.33 mg/L; controls); Cholesterol (213 ± 44 mg/dL; SSNHL vs. 175 ± 21.43 mg/dL controls); LDL (131 ± 32.40 mg/dL SSNHL vs. 110.82 ± 22.66 controls); Nervonic acid (54.38 μmol/L SSNHL vs. 110.07 μmol/L controls). Kassner et al. [13] reported significant differences in values of cd40L (5.12 ± 1.15 ng/mL SSNHL vs. 1.42 ± 0.41 ng/mL control) and L (1.43 ± 0.2 SSNHL vs. 2.23 ± 0.2 control). Significant differences in terms of ESR (9.54 ± 6.66 in the control group and 16.12 ± 16.83 in the SSNHL); ANA (Original data not reported, only significant differences between groups at *p* = 0.01); and C3 (155.14 ± 49.52 in the control group vs. 126.29 ± 31.53 in the case group) and C4 factor (26.22 ± 11.01 in the control group vs. 36.15 ± 8.98 in the case group) were described by Baradaranfar et al. [21]. Procalcitonin (0.057 ± 0.025 μg/L SSNHL group vs. 0.041 ± 0.016 μg/L control group) [11], NLR (6.10 ± 3.31 SSNHL group vs. 2.24 ± 0.99 control group) [16] and CD86 [20] were also reported as significantly different between SSNHL patients and control groups.

#### 3.3.2. Treatment, Outcome, and Prognosis

Outcome was reported by 9 groups: 7 of them reported treatment in detail, generally consisting of steroids in different doses and various vasoactive substances and integrators [12,14,15,16,17,19,23], while two authors did not report treatment modality [11,22]. Treatment results were analyzed according to Siegel’s criteria [23] by 2 groups [12,17]. Siegel’s Criteria is a clinical standardized and widely used criteria for evaluating the outcome of SSNHL. It aims to define the absolute hearing gain after treatment: the difference between Pure Tone Audiometry values at diagnosis and follow-up aim to classify as no recovery, slight recovery, partial recovery, and complete recovery [12]. Seven groups used other criteria to report outcome. Overall, the outcomes ranged from a minimum [11] of 52% to a maximum [23] of 78% of improved patients.

Comprehensively, 7 groups reported significant differences in biomarkers’ values terms between recovered and non-recovered SSNHL patients, as summarized in Table 2 [11,12,16,17,19,22,23]. Two studies [22,23] found significant differences in TNF-α values: 24.1 ± 3.7 pg/mL in the recovered group and 27.6 ± 3.7 pg/mL in the non-recovered group [22]. Demirhan et al. [23] reported that there was no significant difference between pre- and post-treatment values of TNF-α in treatment responders, whereas treatment non-responders had higher post-treatment values of TNF-α than pretreatment values. Exact values were not reported by the authors. CRP and CAR were reported as significant prognostic biomarkers by two investigations: Guo et al. [16] did not report absolute values, whereas Cayr et al. [17] reported CAR 0.73 (range 0.19–1.64) in the recovered group vs. 0.89 (range 0.25–2.19) in the non-recovered one. This last study also found a significant difference of NLR (2.0 range 1.63–3.5 vs. 2.6 range 1.5–3.3) and FAR (69.96 (range 43.03–134.84) vs. 85.22 range (64.65–139.02)) in recovered vs. non-recovered patients [17]. Prognostically, Masuda et al. [19] reported significant differences of neutrophil (<3912/µL recovered vs. >4963/µL non-recovered patients), IL-6 (1.1 ± 0.60 pg/mL patients with normal neutrophil count vs. 2.36 ± 0.83 pg/mL patients with abnormal neutrophil count), and NKCA (20% recovered vs. 33% non-recovered patients).

### 3.4. Quantitative Synthesis

**Figure 2:**
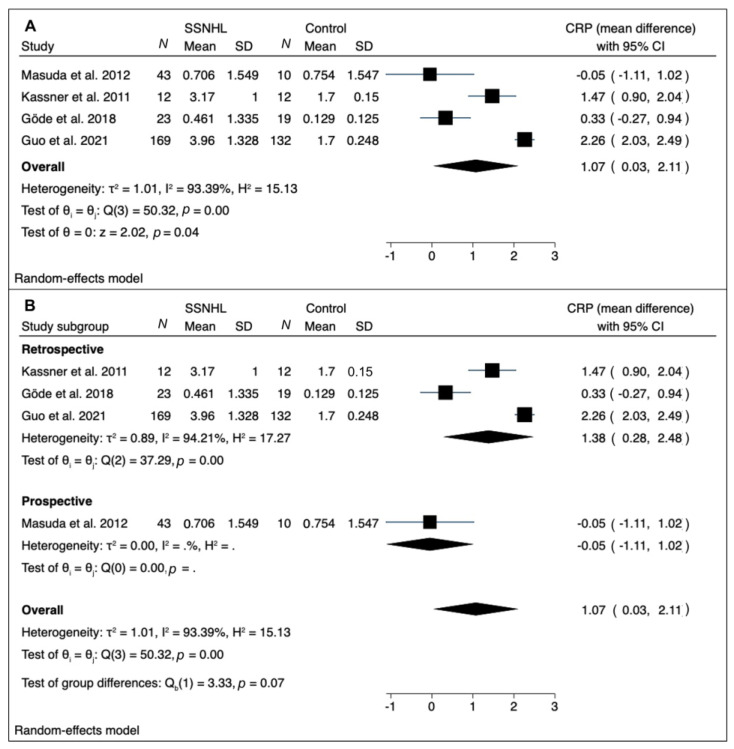
(**A**) Forest plot of mean differences for CRP levels in SSNHL patients vs. controls; (**B**) forest plot showing subgroup analysis by study design (retrospective vs. prospective). Heterogeneity assessed by τ^2^, I^2^, and H^2^ statistics. Abbreviations: SSNHL: sudden sensorineural hearing loss; *N*: number; SD: standard deviation; CRP: C reactive protein.

**Figure 3:**
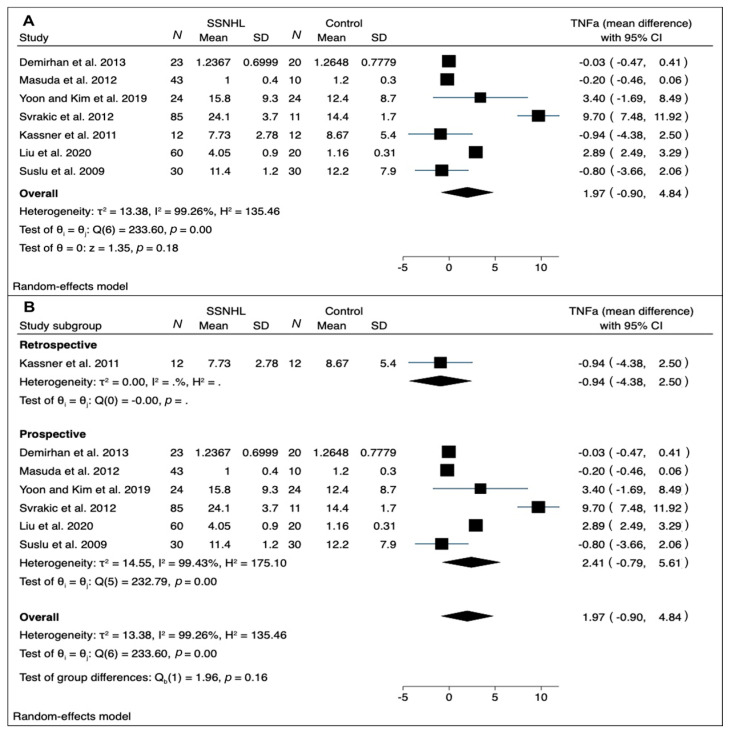
(**A**) Forest plot of WMD for TNF-α levels in SSNHL groups vs. control ones; (**B**) forest plot showing subgroup analysis by study design (retrospective vs. prospective). Heterogeneity assessed by τ^2^, I^2^, and H^2^ statistics. Abbreviations: SSNHL, sudden sensorineural hearing loss; *N*, number; SD, standard deviation; TNF-α, tumor necrosis factor-alpha.

A meta-analysis was performed of mean difference values of CRP [11,13,16,19] and TNF-α [13,14,15,19,20,22,23] in SSNHL and healthy controls (Figure 2 and Figure 3).

The results of our meta-analysis for CRP mean values in SSNHL patients vs. control groups are depicted in Figure 2. The RE model showed significantly higher CRP levels in the pooled overall difference analysis in SSNHL patients compared to controls (pooled mean difference: 1.07; confidence interval at 95%: 0.03; 2.11): a high between-study heterogeneity was found (I^2^ = 93.39%).

A subgroup analysis by study design (retrospective vs. prospective) led to the identification of only one article with prospective design [20]; the remaining retrospective studies confirmed significantly higher CRP levels in SSNHL patients vs. control with a high residual heterogeneity (I^2^ = 94.21%) (Figure 2B). To further investigate heterogeneity, a meta-regression was performed, considering age and gender as moderators. None of these variables reached statistical significance (*p* = 0.162, and *p* = 0.659, respectively), and a significant residual heterogeneity persisted (I^2^ = 94.01%; test of residual homogeneity: *p* < 0.001).

Figure 3 shows the results of meta-analysis for TNF-α. The RE model for TNF-α disclosed discordant results: three studies showed significantly higher levels in SSNHL patients vs. control ones [15,20,22]; the other three investigations showed lower levels in the SSNHL groups [13,14,19]. The overall pooled mean difference was 1.97 (95% CI, −0.90; 4.84). A high between-study heterogeneity was found (I^2^ = 99.26%).

A subgroup analysis by study design (retrospective vs. prospective) led to the identification of only one article with retrospective design [13]; the remaining prospective studies confirmed the trend toward higher TNF-α levels in SSNHL patients vs. control with a high residual heterogeneity (I^2^ = 99.43%) (Figure 3B). A meta-regression found that neither age nor gender reached statistical significance (*p* = 0.835, and *p* = 0.776, respectively), and a significant residual heterogeneity persisted (I^2^ = 98.42%; test of residual homogeneity: *p* < 0.001).

With regard to prognostic values of biomarkers, it was not possible to perform a quantitative synthesis due to paucity of data: only seven out of thirteen research groups reported the outcome data, with heterogeneous markers included (see Table 2). Moreover, two research groups did not detail the absolute data, as previously mentioned [16,23].

## 4. Discussion

### 4.1. The Reason for Investigating Biomarkers in SSNHL

SSNHL is a challenging clinical entity that counts about 66,000 new cases per year in the USA, the majority of which result as idiopathic [1]. If not recognized promptly and managed adequately, it can result in persistent hearing loss that inevitably affects patient quality of life. Moreover, the possible impact on individual social life and cognitive functioning—due to the well-known pleiotropic role of auditory pathway on working memory, attention, and cognitive process—has been highlighted by the evidence that hearing loss has been found to be a major risk factor for dementia [24]. The social cost of SSNHL audiological treatment is not negligible: its annual economic cost is estimated at $750 billion globally, and a recent study found that novel targeted therapeutics for SSNHL could have potential value in terms of both improved patient outcomes and incremental net monetary, with a saving of £6805 (£4875–8736), and an increment in Quality Adjusted Life years (QALYs) of 1.61 (0.79–2.43) per patient per year [25].

These aspects explain the importance of studying markers of SSNHL to help unravel the differential etiopathogenetic diagnosis between viral, immune-mediated and ischemic hypotheses, and consequently choose, dose, and monitor the best individualized therapy [1].

### 4.2. The Role of Circulating Biomarkers in SSNHL

At the current state of the art, the strongest evidence has been produced in regard to peripheral immune system cells such as Neutrophil count, Lymphocyte count, NLR, and PLR. The elevation of these parameters has been reported to correlate with poor prognosis in SSNHL patients [4,5,6].

In recent years, several studies have aimed to establish the role of circulating biomarkers of possible clinical utility for SSNHL management. Following our inclusion and exclusion criteria, 13 studies were analyzed in this systematic review. Overall, we found a low quality of evidence in available studies, with only one investigation rated 9 stars according to the NOS scale [15], consisting of a high-quality study (see Table 1). The main issues were poor description of biomarkers laboratory analysis [12,16,17,20,21] and inadequate selection of control groups for all the included studies. Moreover, we found lack of information and heterogeneity regarding time intervals elapsed from the onset of SSNHL to blood sample collections. These possible biases in study design could have led to an underestimation and/or misinterpretation of biomarkers role and significance. Nonetheless, 11 out of 13 studies reported at least one statistically significant higher value of analyzed biomarkers between SSNHL patients and non-SSNHL controls. Excluding circulating inflammatory cells, as their role in SSNHL diagnosis and prognosis has been discussed earlier [4,5,6], the main reported biomarkers by our included studies were CRP [11,12,13,16,17,19], TNF-α [13,14,15,19,20,22,23], and monocytes [19,20,21]. After being activated by pro-inflammatory cytokines, local growth factors, and microbial products, monocytes migrate into tissues, and can differentiate into a range of tissue dendritic cells or macrophages. We found 3 investigations that reported higher values of monocytes in the study groups [19,20,21]. In particular, Yoon et al. [20] showed that the monocyte population percentage was significantly higher in the 24 patients of the SSNHL group than in the controls one without clinical history of SSNHL. Moreover, these authors identified the presence of dendritic cell markers, namely, clusters of differentiation 11c and 86; the cluster of differentiation 86 level was significantly higher in the SSNHL group than in the control one. It was suggested that TNF-α can enhance the differentiation of monocytes into mature dendritic cells and further studies could clarify the role of dendritic cells in SSNHL [20]. Further studies are also needed before performing a meaningful meta-analysis of the role of monocytes in SSNHL.

### 4.3. Analyzing CRP and TNF-α Trends

Regarding CRP, significantly higher levels in the pooled overall difference analysis in SSNHL compared to controls (pooled difference: 1.07; confidence interval at 95%: 0.03; 2.11) were retrieved. This meta-analytic result, however, may not be considered conclusive regarding the role of CRP in SSNHL at time of diagnosis, given the null results of two singular studies [11,19], and the markedly elevated heterogeneity levels identified on the pooled analysis, which could not be reduced even after subgroup analysis and meta-regression. The reasons for such a large heterogeneity may not be explained just by the study design and patients’ demographics (age and gender), as ruled out by subgroup analysis and meta-regression, but probably reside in pre-analytical and analytical issues related with CRP evaluation. First of all, the blood sampling time plays a critical role. As shown in Table 2, in all the considered studies, blood samples were collected before starting steroid treatment, thus ruling out the effect of therapy on the evaluated inflammatory markers. However, most of the included articles did not exactly report the time interval between symptom onset, hospital admission, and blood collection, leaving a certain degree of uncertainty on this issue, which may partly explain the heterogeneity. Moreover, the inter-laboratory differences may account for a part of the heterogeneity, which may be hardly quantified.

Regarding the prognostic value of CRP, one study disclosed statistically significant differences in good prognosis vs. poor prognosis SSNHL patients [16], whereas two investigations found no statistically significant differences [11,19]. This high heterogeneity of results, which does not clearly indicate the relation of CRP levels and SSNHL clinical course, needs to be stressed once again. CRP is the classical acute phase reactant, and the circulating concentration rises rapidly in a cytokine-mediated response to tissue injury, infection, and inflammation. Blood CRP values are routinely measured to detect and monitor many human diseases. Metabolic functions of CRP are exerted through its capacity for binding to exogenous and autologous molecules containing phosphocholine and then activating the classical complement pathway [26]. In this regard, Baradaranfar et al. [21] found significantly different levels of C3 and C4 components in 56 SSNHL patients vs. normal hearing controls.

CRP/Albumine ratio can be considered as a more functional marker, as hypoalbuminemia is a chronic malnutrition indicator, and combining elevated acute inflammation and decreased serum albumin may indicate nutritional deficiency and poor patient performance, which could all influence the prognosis, especially in the oncology field [27]. Two investigations proposed low CAR levels as good prognostic markers for SSNHL [15,16], while Öçal et al. [12] found non-significant differences.

In the past decades, the role of circulating levels of CRP in the risk of cardiovascular disease and ictus has been established [16]. From this evidence, researchers postulated a possible role of CRP in SSNHL that, according to the results of our review, is reasonable, but needs further investigations (preferably in a prospective multi-center setting), due to the overall low quality of the available evidence and their high heterogeneity.

Considerations similar to these regarding CRP could also be applied to our meta-analysis results about TNF-α. In particular, the overall pooled analysis failed to find statistically significant differences in TNF-α comparing SSNHL cases and controls, but a trend toward higher values in patients was found. Additionally, in this case, subgroup analysis and meta-regression could not explain the very high overall heterogeneity just with study design and demographics, with most of the heterogeneity probably due to the above-mentioned pre-analytical and analytical issues.

TNF-α is a pleiotropic cytokine, a regulatory factor of the immune system that also plays a role in the homeostasis process in a wide range of disorders. It is produced by various cells, but its primary source is that of the immune system (e.g., macrophages, lymphoid cells, mast cells) from which the cytokine is released as soluble TNF-α. Once released, it can bind to TNF-α receptor type 1, which plays a role in apoptosis and the cell survival process, or TNF-α receptor type 2, which contributes to immune response and inflammation. By activating many different intracellular signaling pathways, TNF-α can ultimately lead to cell survival, cell migration, apoptosis, and necrosis [28]. For TNF-α, our meta-analysis disclosed highly discordant results, with an overall pooled difference of 0.27 (95% CI, −0.65; 1.19) from SSNHL and normal hearing controls (see Figure 3). Similarly, two studies found significantly higher values of TNF-α in patients with favorable prognosis compared to those with poor prognosis [22,23]. On the other hand, two investigations found no significant differences, concluding no prognostic value of this biomarker [14,19]. In the inner ear, it has been shown that TNF-α is expressed by many infiltrated cells very early in the inflammatory response in mice and plays a role in reducing spiral modiolar artery blood flow [29,30]. These data supported the use of anti-TNF-α drugs in the therapy of SSNHL of autoimmune origin such as Cogan syndrome [31]. According to Svrakic et al. [22], plasma TNF-α levels higher than 18.3 pg/mL may confirm an immune-mediated pathogenesis in SSNHL with 97% certainty; this high specificity, however, comes at a cost of very low sensitivity, so the researchers concluded that plasmatic TNF-α cannot be used as a screening test, but rather as a confirmatory test. In contrast, Suslu et al. [14] found significantly lower TNF-α values in their group of 30 SSNHL patients. These results could be explained by peri-saccular fibrosis due to chronic inflammation of the endolymphatic sac causing a reduction in number of macrophages and lymphocytes and consequently the reduction of TNF-α release. This pathophysiological hypothesis, although interesting, was derived from animal studies on Meniere’s disease, therefore its applicability for SSNHL needs to be confirmed. With regard to TNF-α pathway, IL-6 and NKFB have been found to have a significant prognostic role in a study on 43 patients with SSNHL compared with 10 patients with non-idiopathic SSNHL (Meniere’s disease, perilymph fistula, and bilateral progressive sensorineural hearing loss) [19]. It was previously demonstrated on an animal model that IL-6 and TNF-α upregulation in the cochlea tissues induced sensorineural hearing loss [32]. Both cytokines have been reported as involved in NF-KB associated cellular stress pathways [19].

### 4.4. Strengths and Limitations of Current Data and Future Prospects

This meta-analysis is affected by several limitations: (1) the presence of retrospective and prospective studies with overall quality mainly affected by scarce quality in control groups selections; (2) the heterogeneity of investigations that aimed to compare many different markers with different biological behavior; (3) the lack of adequate report of outcome and/or inadequate report of biomarkers levels that prevented meta-analysis of data regarding prognostic values of CRP and TNF-α. Due to these limitations, we could not establish a certain role of circulating biomarkers in SSNHL, but we gave an overview of current knowledge, especially regarding the role of the most studied biomarkers such as CRP and TNF-α. Moreover, we pointed out that the growing literature in this field and the results of this review suggest the need for proceeding into digging out the role of circulatory biomarkers in SSNHL. Future well-designed investigations conducted following international guidelines for SSNHL [1], adequate collecting of and reporting biomarkers levels, and using standardized methods of outcome measures could give clinicians further instruments of prognostic and therapeutic utility in this still-challenging disorder.

## 5. Conclusions

In the clinical research of inflammatory biomarkers in SSNHL, CRP, and TNF-α were revealed as the most studied circulating factors. CRP appeared to be higher in SSNHL patients than in controls, while TNF-α showed more heterogeneous behavior and could play a role in SSNHL of immune-mediated etiology, as reputed by the current state of the art. The prognostic role of CRP and TNF-α remains to be clarified. The data reported herein need to be confirmed in well-designed future studies, with the objective of improving SSNHL treatment and outcome and therefore reducing the social burden of this disorder.

## Figures and Tables

**Table 1:** Assessment of quality of included studies according to the Newcastle–Ottawa Scale [10].

	Study Type	Country	Selection				Comparability	Exposure			
Study			Adequacy	Represen- tativeness	Selection	Definition		Ascertainment	Same method	Non-response rate	Total (9/9)
Göde et al. 2018 [11]	Observational retrospective case-control	Turkey	*	*		*	*	*			5
Öçal et al. 2020 [12]	Longitudinal retrospective case-control	Turkey	*	*		*	*	*			5
Kassner et al. 2011 [13]	Observational retrospective case-control	Germany	*	*			**	*			5
Cayir et al. 2021 [17]	Cross-sectional retrospective case-control	Turkey	*	*	*	*	**	*		*	8
Guo et al. 2021 [16]	Observational retrospective case-control	China	*	*		*	**	*			6
Cadoni et al. 2010 [18]	Observational prospective case-control	Italy	*	*	*	*	**	*	*		8
Yoon et al. 2018 [20]	Observational prospective case-control	South Korea	*	*		*	*	*			5
Svakic et al. 2012 [22]	Longitudinal prospective case-control	USA	*		*	*	*	*			5
Baradaranfar et al. 2018 [21]	Observational prospective case-control	Iran	*	*			**	*			5
Demirhan et al. 2013 [23]	Prospective clinical trial	Turkey	*	*			*	*		*	5
Suslu et al. 2009 [14]	Prospective clinical trial	Turkey	*	*		*	**	*			6
Masuda et al. 2012 [19]	Observational longitudinal prospective cohort	Japan	*	*			**	*	*	*	7
Liu et al. 2020 [15]	Observational prospective case-control	China	*	*	*	*	**	*	*	*	9

Legend: * A maximum of one ‘star’ for each item within the ‘Selection’ and ‘Exposure’ categories; maximum of two ‘stars’ for ‘Comparability’, according to the NOS score [10].

**Table 2:** Reported inflammatory biomarkers values and statistical significance in the included studies.

Study	Years of Age (Mean and Standard Deviation)	Sex (Male/Female)	Time Intervals Elapsed from the Onset of SSNHL to Blood Sampling	Diagnostic Value (*p* < 0.05 SSNHL vs. Control Group)	Prognostic Value (*p* < 0.05 Good Prognosis vs. Poor Prognosis)
Cayir et al. 2020 [17]	42.29 ± 9.2	24/23	<5 days; blood sampling on the first day of admission, before corticosteroid treatment	**CAR; FAR; NLR**; PLR; Hgb; WBC	**CAR; FAR; NLR**; PLR; Hgb; WBC
Öçal et al. 2020 [12]	44.1 ± 14.2	24/16	Admission time	**CAR**	CAR; NLR
Masuda et al. 2012 [19]	57 ± 15	23/20	<7 days; blood sampling at the first visit, before corticosteroid treatment	TNF-α; CRP; N; L; **M**; IL-6; NKCA	TNF-α; CRP; **N**; L; M; **IL-6; NKCA**
Liu et al. 2020 [15]	ND	41/79	ND	**TNF-α;** **TLR-4; NFKB**	ND
Kassner et al. 2011 [13]	45.4 ± 4.1	ND	6–24 h; blood sampling before corticosteroid treatment	TNF-α; CRP; WBC; N; **L**; E; B; LDL; HDL; **CD40**	ND
Cadoni et al. 2010 [18]	50 ± 14	19/24	Admission time	**Q10; Cholesterol; LDL; Nervonic acid**	ND
Yoon and Kim et al. 2019 [20]	46.91 ± ND	15/9	Before corticosteroid treatment	**TNF-α; M; CD86**	ND
Baradaranfar et al. 2018 [21]	40.80 ± 13.37	26/30	ND	**ESR**; Anti CCP; Anti-dsDNA; **ANA; C3 and C4**; WBC; N; L; E; P; **M**	ND
Svrakic et al. 2012 [22]	52.51 ± 16.08	35/50	Before corticosteroid treatment	**TNF-α**	**TNF-α**
Göde et al. 2018 [11]	47.91 ± 15.73	14/0	Before corticosteroid treatment	CRP; **Procalcitonin**	CRP; Procalcitonin
Demirhan et al. 2013 [23]	52 ± ND	13/10	Before corticosteroid treatment	TNF-α; IL-10; IL-12	**TNF-α**; IL-10; IL-12
Guo et al. 2021 [16]	46.69 ± 16.76	80/89	Before corticosteroid treatment	CRP; CAR; **NLR**	**CRP; CAR**; NLR
Suslu et al. 2009 [14]	42 ± ND	11/19	ND	ESR; **TNF-α**; Anti HSP-70; ANA	ESR; TNF-α; Anti HSP-70; ANA

Legend: Statistically significant markers are in bold. Abbreviations: ANA: antinuclear antibody; Anti CCP: anti-cyclic citrullinated peptide; Anti-dsDNA: Anti-double stranded DNA Antibodies; Anti HSP-70: Anti-Heat shock protein 70; B: B lymphocytes; C3: complement component 3; C4: complement component 4; CAR: C-reactive protein/albumin ratio; CD40: cluster of differentiation 40; CD86 cluster of differentiation 86; CRP: C- reactive protein; E: eosinophil; ESR: erythrocyte sedimentation rate; FAR: fibrinogen/albumin ratio; HDL: high-density lipoprotein; Hgb: Hemoglobin; IL-6: Interleukin-6; IL-10: Interleukin-10; IL-12: Interleukin-12; L: lymphocyte; LDL: low-density lipoprotein; M: monocyte; N: neutrophil; ND: No Data; NKCA: natural killer cell activity; NFKB: nuclear factor kappa-light chain-enhancer of activated B cells; Neutrophil to Lymphocyte ratio (NLR) [11,15,16]; P: platelets; PLR: Platelet/Lymphocyte Ratio; Q10: Coenzyme Q10; TLR-4: Toll like receptor-4; TNF-α: tumor necrosis factor; WBC: white blood cells.

## Data Availability

The data presented in this study are available on request from the corresponding author.
